# Ethyl 1-*tert*-butyl-5-phenyl-1*H*-pyrazole-4-carboxyl­ate

**DOI:** 10.1107/S1600536810030643

**Published:** 2010-08-11

**Authors:** Hoong-Kun Fun, Ching Kheng Quah, B. Chandrakantha, Arun M. Isloor, Prakash Shetty

**Affiliations:** a X-ray Crystallography Unit, School of Physics, Universiti Sains Malaysia, 11800 USM, Penang, Malaysia; b Department of Chemistry, Manipal Institute of Technology, Manipal 576 104, India; c Organic Chemistry Division, Department of Chemistry, National Institute of Technology–Karnataka, Surathkal, Mangalore 575 025, India; d Department of Printing, Manipal Institute of Technology, Manipal 576 104, India

## Abstract

In the title compound, C_16_H_20_N_2_O_2_, the pyrazole ring is essentially planar [maximum deviation = 0.008 (2) Å] and is inclined at an angle of 82.82 (10)° with respect to the phenyl ring. The crystal packing is consolidated by pairs of inter­molecular C—H⋯O hydrogen bonds, which link the mol­ecules into centrosymmetric dimers stacked along the *a* axis.

## Related literature

For general background to pyrazole derivatives and their biological activity, see: Isloor *et al.* (2009 ▶); Lambert & Fowler (2005 ▶); Lan *et al.* (1999 ▶). For related structures, see: Fun *et al.* (2009 ▶; 2010*a* ▶,*b* ▶). For bond-length data, see: Allen *et al.* (1987 ▶). For the stability of the temperature controller used in the data collection, see: Cosier & Glazer (1986 ▶).
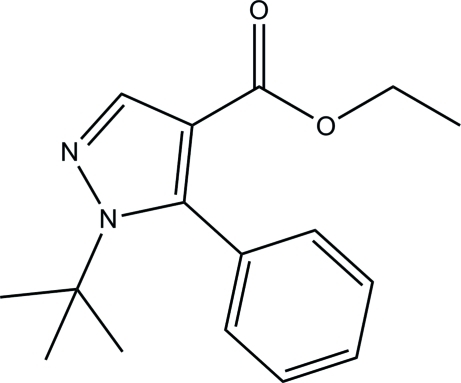


## Experimental

### 

#### Crystal data

C_16_H_20_N_2_O_2_
*M* *_r_* = 272.34
Triclinic, 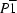

*a* = 9.0665 (2) Å
*b* = 9.3351 (2) Å
*c* = 10.5408 (3) Å
α = 110.450 (1)°
β = 113.987 (1)°
γ = 97.645 (2)°
*V* = 723.22 (3) Å^3^
*Z* = 2
Mo *K*α radiation
μ = 0.08 mm^−1^
*T* = 100 K
0.41 × 0.20 × 0.12 mm

#### Data collection

Bruker SMART APEXII CCD area-detector diffractometer
Absorption correction: multi-scan (*SADABS*; Bruker, 2009 ▶) *T*_min_ = 0.967, *T*_max_ = 0.990
12406 measured reflections
2655 independent reflections
2179 reflections with *I* > 2σ(*I*)
*R*_int_ = 0.038

#### Refinement

*R*[*F* ^2^ > 2σ(*F* ^2^)] = 0.042
*wR*(*F* ^2^) = 0.099
*S* = 1.04
2655 reflections
185 parameters
H-atom parameters constrained
Δρ_max_ = 0.22 e Å^−3^
Δρ_min_ = −0.20 e Å^−3^

### 

Data collection: *APEX2* (Bruker, 2009 ▶); cell refinement: *SAINT* (Bruker, 2009 ▶); data reduction: *SAINT*; program(s) used to solve structure: *SHELXTL* (Sheldrick, 2008 ▶); program(s) used to refine structure: *SHELXTL*; molecular graphics: *SHELXTL*; software used to prepare material for publication: *SHELXTL* and *PLATON* (Spek, 2009 ▶).

## Supplementary Material

Crystal structure: contains datablocks global, I. DOI: 10.1107/S1600536810030643/ci5147sup1.cif

Structure factors: contains datablocks I. DOI: 10.1107/S1600536810030643/ci5147Isup2.hkl

Additional supplementary materials:  crystallographic information; 3D view; checkCIF report

## Figures and Tables

**Table 1:** Hydrogen-bond geometry (Å, °)

*D*—H⋯*A*	*D*—H	H⋯*A*	*D*⋯*A*	*D*—H⋯*A*
C15—H15*B*⋯O2^i^	0.97	2.53	3.367 (2)	145

Symmetry code: (i) 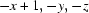.

**Fig. 1.**
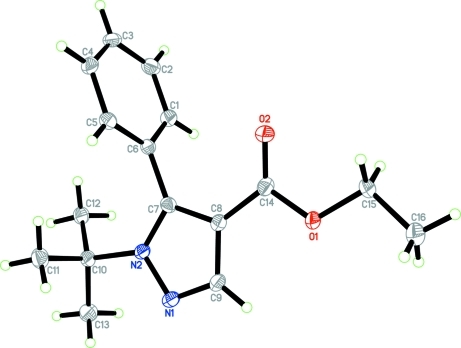
The molecular structure of the title compound, showing 50% probability displacement ellipsoids for non-H atoms and the atom-numbering scheme.

**Fig. 2.**
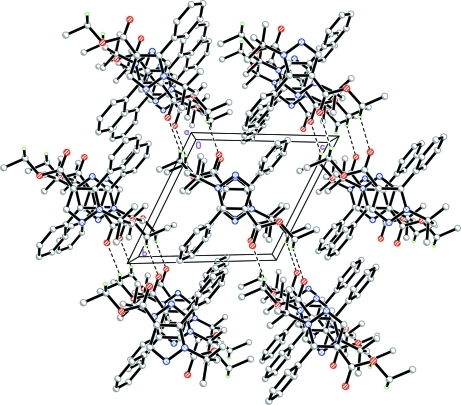
The crystal structure of the title compound, viewed along the a axis. H atoms not involved in hydrogen bonds (dashed lines) have been omitted for clarity.

## supplementary crystallographic information

### Comment

Pyrazole and its derivatives represent one of the most active classes of compounds possessing a wide spectrum of biological activities. During the past years, considerable evidences have been accumulated to demonstrate the efficacy of pyrazole derivatives including antibacterial (Isloor *et al.*, 2009), antifungal (Lambert & Fowler, 2005), herbicidal (Lan *et al.*, 1999), insecticidal and other biological activities. Keeping in view of the importance of the pyrazole derivatives, we have synthesized a new pyrazole molecule, with the aim of studying its single crystal structure.

The title molecule (Fig. 1) consists of a phenyl ring (C1-C6), a tert-butyl moiety (C10-C13) and a ethyl carboxylate moiety (O1/O2/C14-C16) attached to a pyrazole ring (N1/N2/C7-C9). The pyrazole ring is essentially planar (maximum deviation = 0.008 (2) Å for atom N2) and is inclined at an angle of 82.82 (10)° with the phenyl ring. Bond lengths and angles are within normal ranges, and comparable to closely related structures (Fun *et al.*, 2009,2010*a*,*b*).

In the crystal structure (Fig. 2), the crystal packing is consolidated by pairs of intermolecular C15—H15B···O2 hydrogen bonds linking the molecules into centrosymmetric dimers which are stacked down the *a* axis.

### Experimental

A mixture of ethyl-3-(dimethylamino)-2-(phenylcarbonyl)prop-2-enoate (2.0 g, 0.0080 mol), tert-butyl hydrazine.HCl (1.09 g, 0.0088 mol) and sodium bicarbonate (2.05 g, 0.0240 mol) in absolute ethanol (25 ml) was refluxed for 2 h. Reaction completion was monitored through thin layer chromatography and the reaction mixture was evaporated under reduced pressure. The residue was stirred with 1.5N HCl and the solid separated was filtered and dried under vacuum. The solid obtained was purified by column chromatography using silica gel 60-120 mesh size and petroleum ether-ethyl acetate as eluent to afford title compound as yellow solid (1.5g, 71%); melting point 343-347 K.

### Refinement

All H atoms were positioned geometrically and refined using a riding model with C-H = 0.93-0.97 Å and *U*_iso_(H) = 1.2 or 1.5 *U*_eq_(C). A rotating-group model was applied for the methyl groups.

### Crystal data

**Table:**

C_16_H_20_N_2_O_2_	*Z* = 2
*M**_r_* = 272.34	*F*(000) = 292
Triclinic, *P*1	*D*_x_ = 1.251 Mg m^−^^3^
Hall symbol: -P 1	Mo *K*α radiation, λ = 0.71073 Å
*a* = 9.0665 (2) Å	Cell parameters from 6289 reflections
*b* = 9.3351 (2) Å	θ = 2.4–34.3°
*c* = 10.5408 (3) Å	µ = 0.08 mm^−^^1^
α = 110.450 (1)°	*T* = 100 K
β = 113.987 (1)°	Needle, yellow
γ = 97.645 (2)°	0.41 × 0.20 × 0.12 mm
*V* = 723.22 (3) Å^3^	

### Data collection

**Table:**

Bruker SMART APEXII CCD area-detector diffractometer	2655 independent reflections
Radiation source: fine-focus sealed tube	2179 reflections with *I* > 2σ(*I*)
graphite	*R*_int_ = 0.038
φ and ω scans	θ_max_ = 25.5°, θ_min_ = 2.4°
Absorption correction: multi-scan (*SADABS*; Bruker, 2009)	*h* = −10→10
*T*_min_ = 0.967, *T*_max_ = 0.990	*k* = −11→11
12406 measured reflections	*l* = −12→12

### Refinement

**Table:**

Refinement on *F*^2^	Primary atom site location: structure-invariant direct methods
Least-squares matrix: full	Secondary atom site location: difference Fourier map
*R*[*F*^2^ > 2σ(*F*^2^)] = 0.042	Hydrogen site location: inferred from neighbouring sites
*wR*(*F*^2^) = 0.099	H-atom parameters constrained
*S* = 1.04	*w* = 1/[σ^2^(*F*_o_^2^) + (0.0402*P*)^2^ + 0.3388*P*] where *P* = (*F*_o_^2^ + 2*F*_c_^2^)/3
2655 reflections	(Δ/σ)_max_ = 0.001
185 parameters	Δρ_max_ = 0.22 e Å^−^^3^
0 restraints	Δρ_min_ = −0.20 e Å^−^^3^

### Special details

**Table:**

Experimental. The crystal was placed in the cold stream of an Oxford Cyrosystems Cobra open-flow nitrogen cryostat (Cosier & Glazer, 1986) operating at 100.0 (1) K.
Geometry. All esds (except the esd in the dihedral angle between two l.s. planes) are estimated using the full covariance matrix. The cell esds are taken into account individually in the estimation of esds in distances, angles and torsion angles; correlations between esds in cell parameters are only used when they are defined by crystal symmetry. An approximate (isotropic) treatment of cell esds is used for estimating esds involving l.s. planes.
Refinement. Refinement of F^2^ against ALL reflections. The weighted R-factor wR and goodness of fit S are based on F^2^, conventional R-factors R are based on F, with F set to zero for negative F^2^. The threshold expression of F^2^ > 2sigma(F^2^) is used only for calculating R-factors(gt) etc. and is not relevant to the choice of reflections for refinement. R-factors based on F^2^ are statistically about twice as large as those based on F, and R- factors based on ALL data will be even larger.

### Fractional atomic coordinates and isotropic or equivalent isotropic displacement parameters (Å^2^)

**Table:**

	*x*	*y*	*z*	*U*_iso_*/*U*_eq_	
O1	0.53056 (13)	0.32289 (13)	0.13622 (12)	0.0190 (3)	
O2	0.52454 (14)	0.14419 (13)	0.23468 (13)	0.0247 (3)	
N1	0.85134 (17)	0.68033 (16)	0.57591 (15)	0.0199 (3)	
N2	0.87235 (16)	0.58254 (15)	0.64872 (15)	0.0169 (3)	
C1	0.8628 (2)	0.18877 (19)	0.56614 (19)	0.0203 (4)	
H1A	0.9334	0.2079	0.5259	0.024*	
C2	0.8544 (2)	0.0604 (2)	0.60345 (19)	0.0223 (4)	
H2A	0.9206	−0.0053	0.5895	0.027*	
C3	0.7477 (2)	0.02988 (19)	0.66145 (19)	0.0216 (4)	
H3A	0.7430	−0.0557	0.6872	0.026*	
C4	0.6484 (2)	0.12657 (19)	0.68093 (19)	0.0209 (4)	
H4A	0.5754	0.1050	0.7183	0.025*	
C5	0.65692 (19)	0.25632 (19)	0.64487 (18)	0.0187 (4)	
H5A	0.5899	0.3213	0.6583	0.022*	
C6	0.76584 (19)	0.28886 (19)	0.58869 (18)	0.0166 (4)	
C7	0.77480 (19)	0.42591 (18)	0.54764 (18)	0.0160 (3)	
C8	0.69038 (19)	0.42220 (19)	0.40266 (18)	0.0166 (4)	
C9	0.7435 (2)	0.58270 (19)	0.42835 (19)	0.0190 (4)	
H9A	0.7070	0.6163	0.3507	0.023*	
C10	0.98019 (19)	0.66369 (19)	0.82223 (18)	0.0178 (4)	
C11	0.8627 (2)	0.6922 (2)	0.89211 (19)	0.0233 (4)	
H11A	0.7959	0.7535	0.8527	0.035*	
H11B	0.9299	0.7508	1.0030	0.035*	
H11C	0.7884	0.5902	0.8646	0.035*	
C12	1.0849 (2)	0.5612 (2)	0.87611 (19)	0.0213 (4)	
H12A	1.1484	0.5365	0.8219	0.032*	
H12B	1.0101	0.4628	0.8548	0.032*	
H12C	1.1623	0.6199	0.9856	0.032*	
C13	1.1020 (2)	0.8262 (2)	0.8701 (2)	0.0232 (4)	
H13A	1.0376	0.8944	0.8428	0.035*	
H13B	1.1678	0.8091	0.8175	0.035*	
H13C	1.1771	0.8768	0.9802	0.035*	
C14	0.57491 (19)	0.2808 (2)	0.25368 (19)	0.0178 (4)	
C15	0.4271 (2)	0.19063 (19)	−0.01920 (18)	0.0204 (4)	
H15A	0.3269	0.1272	−0.0275	0.024*	
H15B	0.4921	0.1206	−0.0422	0.024*	
C16	0.3759 (2)	0.2633 (2)	−0.1304 (2)	0.0261 (4)	
H16A	0.3137	0.1788	−0.2348	0.039*	
H16B	0.4759	0.3313	−0.1164	0.039*	
H16C	0.3051	0.3263	−0.1112	0.039*	

### Atomic displacement parameters (Å^2^)

**Table:**

	*U*^11^	*U*^22^	*U*^33^	*U*^12^	*U*^13^	*U*^23^
O1	0.0228 (6)	0.0165 (6)	0.0116 (6)	0.0008 (5)	0.0054 (5)	0.0054 (5)
O2	0.0322 (6)	0.0163 (7)	0.0189 (7)	−0.0002 (5)	0.0098 (5)	0.0070 (5)
N1	0.0249 (7)	0.0164 (7)	0.0176 (8)	0.0037 (6)	0.0089 (6)	0.0094 (6)
N2	0.0197 (7)	0.0145 (7)	0.0143 (7)	0.0020 (6)	0.0070 (6)	0.0070 (6)
C1	0.0222 (8)	0.0189 (9)	0.0181 (9)	0.0033 (7)	0.0100 (7)	0.0075 (7)
C2	0.0250 (8)	0.0169 (9)	0.0211 (9)	0.0067 (7)	0.0085 (8)	0.0076 (7)
C3	0.0262 (8)	0.0142 (9)	0.0164 (9)	0.0001 (7)	0.0043 (7)	0.0080 (7)
C4	0.0222 (8)	0.0193 (9)	0.0156 (9)	−0.0014 (7)	0.0075 (7)	0.0069 (7)
C5	0.0191 (8)	0.0159 (9)	0.0159 (9)	0.0021 (7)	0.0069 (7)	0.0047 (7)
C6	0.0179 (7)	0.0134 (8)	0.0098 (8)	−0.0009 (6)	0.0022 (6)	0.0035 (6)
C7	0.0163 (7)	0.0142 (9)	0.0161 (8)	0.0028 (6)	0.0088 (7)	0.0049 (7)
C8	0.0185 (8)	0.0170 (9)	0.0151 (8)	0.0046 (7)	0.0088 (7)	0.0077 (7)
C9	0.0227 (8)	0.0189 (9)	0.0153 (9)	0.0044 (7)	0.0082 (7)	0.0095 (7)
C10	0.0192 (8)	0.0158 (9)	0.0124 (8)	0.0013 (7)	0.0057 (7)	0.0039 (7)
C11	0.0243 (8)	0.0228 (10)	0.0180 (9)	0.0039 (7)	0.0099 (7)	0.0061 (8)
C12	0.0230 (8)	0.0194 (9)	0.0140 (9)	0.0025 (7)	0.0051 (7)	0.0059 (7)
C13	0.0220 (8)	0.0198 (9)	0.0189 (9)	0.0001 (7)	0.0058 (7)	0.0067 (7)
C14	0.0182 (7)	0.0201 (9)	0.0168 (9)	0.0050 (7)	0.0093 (7)	0.0095 (7)
C15	0.0224 (8)	0.0170 (9)	0.0135 (9)	0.0013 (7)	0.0059 (7)	0.0034 (7)
C16	0.0282 (9)	0.0258 (10)	0.0173 (9)	0.0050 (8)	0.0076 (8)	0.0079 (8)

### Geometric parameters (Å, °)

**Table:**

O1—C14	1.3511 (18)	C8—C14	1.467 (2)
O1—C15	1.4550 (18)	C9—H9A	0.93
O2—C14	1.2116 (18)	C10—C12	1.524 (2)
N1—C9	1.320 (2)	C10—C11	1.524 (2)
N1—N2	1.3695 (17)	C10—C13	1.530 (2)
N2—C7	1.363 (2)	C11—H11A	0.96
N2—C10	1.502 (2)	C11—H11B	0.96
C1—C2	1.388 (2)	C11—H11C	0.96
C1—C6	1.391 (2)	C12—H12A	0.96
C1—H1A	0.93	C12—H12B	0.96
C2—C3	1.386 (2)	C12—H12C	0.96
C2—H2A	0.93	C13—H13A	0.96
C3—C4	1.380 (2)	C13—H13B	0.96
C3—H3A	0.93	C13—H13C	0.96
C4—C5	1.392 (2)	C15—C16	1.499 (2)
C4—H4A	0.93	C15—H15A	0.97
C5—C6	1.393 (2)	C15—H15B	0.97
C5—H5A	0.93	C16—H16A	0.96
C6—C7	1.489 (2)	C16—H16B	0.96
C7—C8	1.388 (2)	C16—H16C	0.96
C8—C9	1.403 (2)		
			
C14—O1—C15	115.77 (12)	C12—C10—C13	108.71 (13)
C9—N1—N2	105.07 (12)	C11—C10—C13	109.39 (14)
C7—N2—N1	111.67 (12)	C10—C11—H11A	109.5
C7—N2—C10	131.06 (13)	C10—C11—H11B	109.5
N1—N2—C10	116.93 (12)	H11A—C11—H11B	109.5
C2—C1—C6	120.21 (15)	C10—C11—H11C	109.5
C2—C1—H1A	119.9	H11A—C11—H11C	109.5
C6—C1—H1A	119.9	H11B—C11—H11C	109.5
C3—C2—C1	120.18 (16)	C10—C12—H12A	109.5
C3—C2—H2A	119.9	C10—C12—H12B	109.5
C1—C2—H2A	119.9	H12A—C12—H12B	109.5
C4—C3—C2	119.92 (15)	C10—C12—H12C	109.5
C4—C3—H3A	120.0	H12A—C12—H12C	109.5
C2—C3—H3A	120.0	H12B—C12—H12C	109.5
C3—C4—C5	120.25 (15)	C10—C13—H13A	109.5
C3—C4—H4A	119.9	C10—C13—H13B	109.5
C5—C4—H4A	119.9	H13A—C13—H13B	109.5
C4—C5—C6	120.01 (15)	C10—C13—H13C	109.5
C4—C5—H5A	120.0	H13A—C13—H13C	109.5
C6—C5—H5A	120.0	H13B—C13—H13C	109.5
C1—C6—C5	119.40 (14)	O2—C14—O1	123.37 (14)
C1—C6—C7	119.91 (14)	O2—C14—C8	126.18 (14)
C5—C6—C7	120.65 (14)	O1—C14—C8	110.45 (13)
N2—C7—C8	106.22 (13)	O1—C15—C16	107.43 (13)
N2—C7—C6	125.57 (14)	O1—C15—H15A	110.2
C8—C7—C6	128.21 (14)	C16—C15—H15A	110.2
C7—C8—C9	105.09 (14)	O1—C15—H15B	110.2
C7—C8—C14	127.70 (14)	C16—C15—H15B	110.2
C9—C8—C14	127.19 (14)	H15A—C15—H15B	108.5
N1—C9—C8	111.93 (14)	C15—C16—H16A	109.5
N1—C9—H9A	124.0	C15—C16—H16B	109.5
C8—C9—H9A	124.0	H16A—C16—H16B	109.5
N2—C10—C12	110.47 (13)	C15—C16—H16C	109.5
N2—C10—C11	108.18 (12)	H16A—C16—H16C	109.5
C12—C10—C11	111.78 (13)	H16B—C16—H16C	109.5
N2—C10—C13	108.24 (12)		
			
C9—N1—N2—C7	−1.56 (17)	C6—C7—C8—C9	−179.96 (15)
C9—N1—N2—C10	−175.57 (13)	N2—C7—C8—C14	177.44 (15)
C6—C1—C2—C3	0.9 (2)	C6—C7—C8—C14	−1.8 (3)
C1—C2—C3—C4	0.5 (2)	N2—N1—C9—C8	1.04 (18)
C2—C3—C4—C5	−1.0 (2)	C7—C8—C9—N1	−0.18 (18)
C3—C4—C5—C6	0.1 (2)	C14—C8—C9—N1	−178.40 (15)
C2—C1—C6—C5	−1.8 (2)	C7—N2—C10—C12	44.2 (2)
C2—C1—C6—C7	−179.70 (15)	N1—N2—C10—C12	−143.23 (13)
C4—C5—C6—C1	1.3 (2)	C7—N2—C10—C11	−78.5 (2)
C4—C5—C6—C7	179.20 (14)	N1—N2—C10—C11	94.12 (15)
N1—N2—C7—C8	1.46 (17)	C7—N2—C10—C13	163.07 (15)
C10—N2—C7—C8	174.38 (15)	N1—N2—C10—C13	−24.31 (18)
N1—N2—C7—C6	−179.31 (14)	C15—O1—C14—O2	−4.6 (2)
C10—N2—C7—C6	−6.4 (3)	C15—O1—C14—C8	175.15 (12)
C1—C6—C7—N2	−97.7 (2)	C7—C8—C14—O2	5.7 (3)
C5—C6—C7—N2	84.4 (2)	C9—C8—C14—O2	−176.47 (16)
C1—C6—C7—C8	81.3 (2)	C7—C8—C14—O1	−174.06 (14)
C5—C6—C7—C8	−96.5 (2)	C9—C8—C14—O1	3.8 (2)
N2—C7—C8—C9	−0.76 (17)	C14—O1—C15—C16	173.80 (13)

### Hydrogen-bond geometry (Å, °)

**Table:**

*D*—H···*A*	*D*—H	H···*A*	*D*···*A*	*D*—H···*A*
C15—H15B···O2^i^	0.97	2.53	3.367 (2)	145

Symmetry codes: (i) −*x*+1, −*y*, −*z*.
